# Genistein Loaded Nanofibers Protect Spinal Cord Tissue Following Experimental Injury in Rats

**DOI:** 10.3390/biomedicines6040096

**Published:** 2018-10-04

**Authors:** Mohamed Ismail, Sara Ibrahim, Azza El-Amir, Amira M. EL-Rafei, Nageh K. Allam, Ahmed Abdellatif

**Affiliations:** 1 Zoology Department, Faculty of Science, Cairo University, Giza 12613, Egypt; dr_mesmail@yahoo.com (M.I.); Azzaelamir@yahoo.com (A.E.-A.); 2 Energy Materials Laboratory, School of Sciences and Engineering, American University in Cairo, New Cairo 11835, Egypt; dms.80270@gmail.com; 3 Supplementary General Science, Faculty of Oral and Dental Medicine, Future University in Egypt, New Cairo 11835, Egypt; 4 Refractories, Ceramics and Building Materials Department, National Research Centre, Dokki, Giza 12622, Egypt; am.amin@nrc.sci.eg; 5 Biology Department, School of Sciences and Engineering, American University in Cairo, New Cairo 11835, Egypt

**Keywords:** genistein, nanofibers, spinal cord injury, inflammation SCI, SOD, NO, MDA, IL-10, TNF-α

## Abstract

Innovative drug-delivery systems offer a unique approach to effectively provide therapeutic drug dose over the needed time to achieve better tissue protection and enhanced recovery. The hypothesis of the current study was to test the antioxidant and anti-inflammatory effects of genistein and nanofibers on the spinal cord tissue following experimental spinal cord injury (SCI). Rats were treated post SCI with genistein that is loaded on chitosan/polyvinyl alcohol (CS/PVA) nanofibers as an implantable drug-delivery system. SCI caused marked oxidative damage and inflammation, as is evident by the reduction in the super oxide dismutase (SOD) activity and the level of interleukin-10 (IL-10) in injured spinal cord tissue, as well as the significant increase in the levels of nitric oxide (NO), malondialdehyde (MDA), and tumor necrosis factor-alpha (TNF-α). Treatment of rats post SCI with genistein and CS/PVA nanofibers improved most of the above-mentioned biochemical parameters and shifted them toward the control group values. Genistein induced an increase in the activity of SOD and the level of IL-10, while causing a decrease in NO, MDA, and TNF-α in injured spinal cord tissue. Genistein and CS/PVA nanofibers provide a novel combination for treating inflammatory nervous tissue conditions, especially when combined as an implantable drug-delivery system.

## 1. Introduction

Central nervous system (CNS) injuries are devastating due to the limited post-injury functional recovery, because of neuronal cell loss and the release of inhibitory substances [1]. The primary mechanical spinal cord injury (SCI) is commonly followed by a secondary phase that is characterized by inflammation, and a cascade of cellular and biological reactions [2,3]. Among these reactions are the activation of inflammatory cascade associated with cytokines and free radical formation and lipid peroxidation, cytokine, and interleukin up regulation around the damaged area [4].

The current consensus is that reducing inflammation may help to decrease secondary damage and the functional deficit following SCI. Standard treatment regimens currently used for nervous system trauma injury include surgery, hypothermia, and pharmaceuticals (e.g., methylprednisolone), which aim at decreasing inflammation and cell after acute injury [5].

Estrogens are used in the treatment of acute SCI for their anti-inflammatory and antioxidant effects, reduction of apoptosis [6], and increasing white-matter sparing [7]. Additionally, rats that were treated with estrogens post SCI showed reduced edema and myelin loss in the lesion [8]. However, long-term treatment in human with estrogen might increase the risk of cancers, especially breast, endometrial, and ovarian cancers [9]. Therefore, other natural compounds with little or no side effects need to be investigated.

Genistein (4′,5,7-Trihydroxyisoflavone) is a natural non-steroidal phytoestrogen extracted from soybean, that influences cellular function by acting as an agonist at estrogen receptor beta (ERβ) [10], which possesses anti-inflammatory [11,12,13] and antioxidant effects [14]. It suppresses tumor necrosis factor-alpha (TNF-α) and decreases the production of reactive oxygen species (ROS), lipid peroxidation, and inhibits the apoptotic signaling cascade [15].

Biomaterial scaffolds can be used to deliver drugs and fill in the cavities that develop as a result of SCI, provide a great potential for CNS repair. To this end, nanofibers can play a significant role in supporting repair after CNS injury. The combination of high porosity, flexibility, and mechanical performance makes such fibers preferred materials for various biomedical applications. Chitosan (CS) is one of the natural polysaccharide polymers, which has unique properties, including anti-inflammatory, antibacterial, antimicrobial effects, in addition to its biocompatibility, biodegradability, renewability, and nontoxicity [16,17,18,19]. Therefore, chitosan has been used in drug delivery systems [20], tissue-engineering applications [21], and in wound healing [22,23,24]. However, due to the limited solubility, and high viscosity of chitosan [25], it is commonly blended with other polymers, such as polyvinyl alcohol (PVA), which is a synthetic biocompatible polymer [26,27,28,29,30].

Towards this goal, we investigated a novel chitosan/polyvinyl alcohol (CS/PVA) nanofiber drug delivery system using genistein as a potential therapeutic agent for the treatment of SCI, due to its anti-inflammatory and antioxidant effects [11].

## 2. Results

### 2.1. Genistein and Nanofibers Increase Super Oxide Dismutase (SOD) Activity in Spinal Cord Tissue

**Figure 1:**
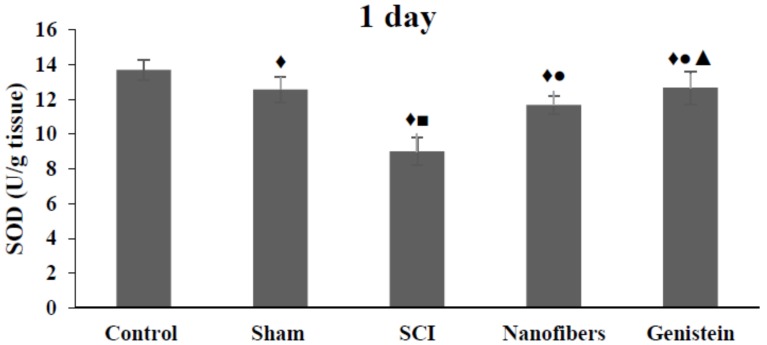

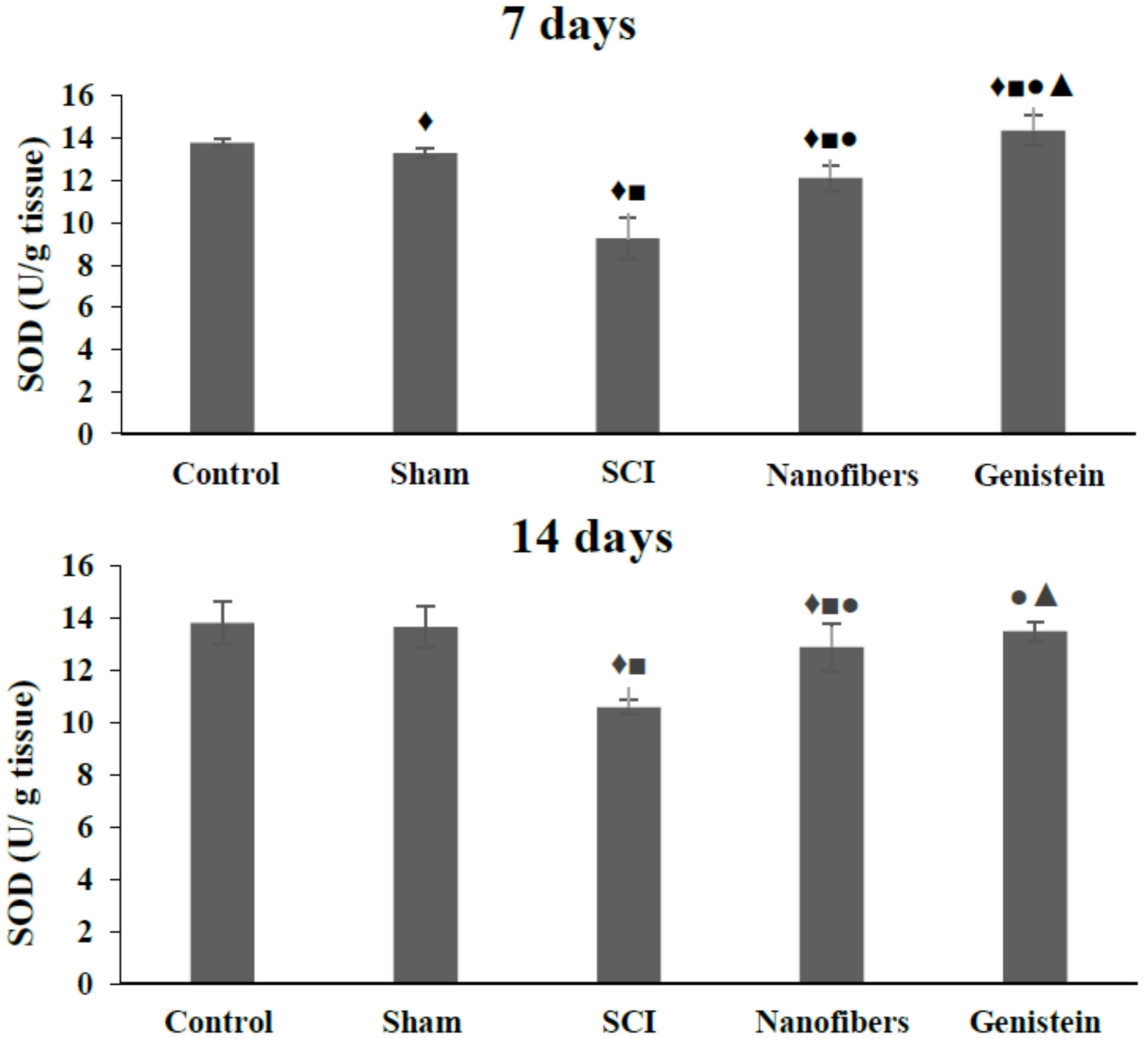
Super oxide dismutase (SOD) activity (U/g tissue) in spinal cord tissue. Genistein & Nanofibers increase super oxide dismutase (SOD) activity in spinal cord tissue of rats in the treatment groups when compared with control (♦) and sham groups (■). Both treatment groups showed significant increase of SOD when compared with SCI group (●, ▲). Number of animals (*n* = 5). Data shown as Mean ± S.E.M.).

The present results show that the activity of SOD was significantly (*p* < 0.05) reduced following SCI when compared to control and sham groups during all of the time intervals studied (Figure 1). The application of nanofibers alone and nanofibers that were loaded with genistein after SCI resulted in a significant (*p* < 0.05) elevation of SOD activity as compared with the SCI group during all of the experimental period.

### 2.2. Genistein Decreases Nitrous Oxide (NO) Concentration in Spinal Cord Tissue

**Figure 2:**
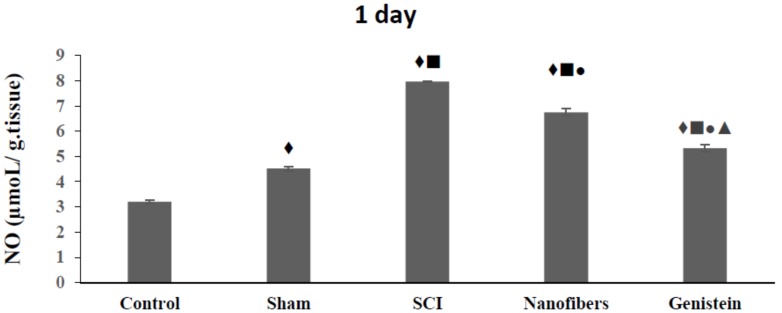

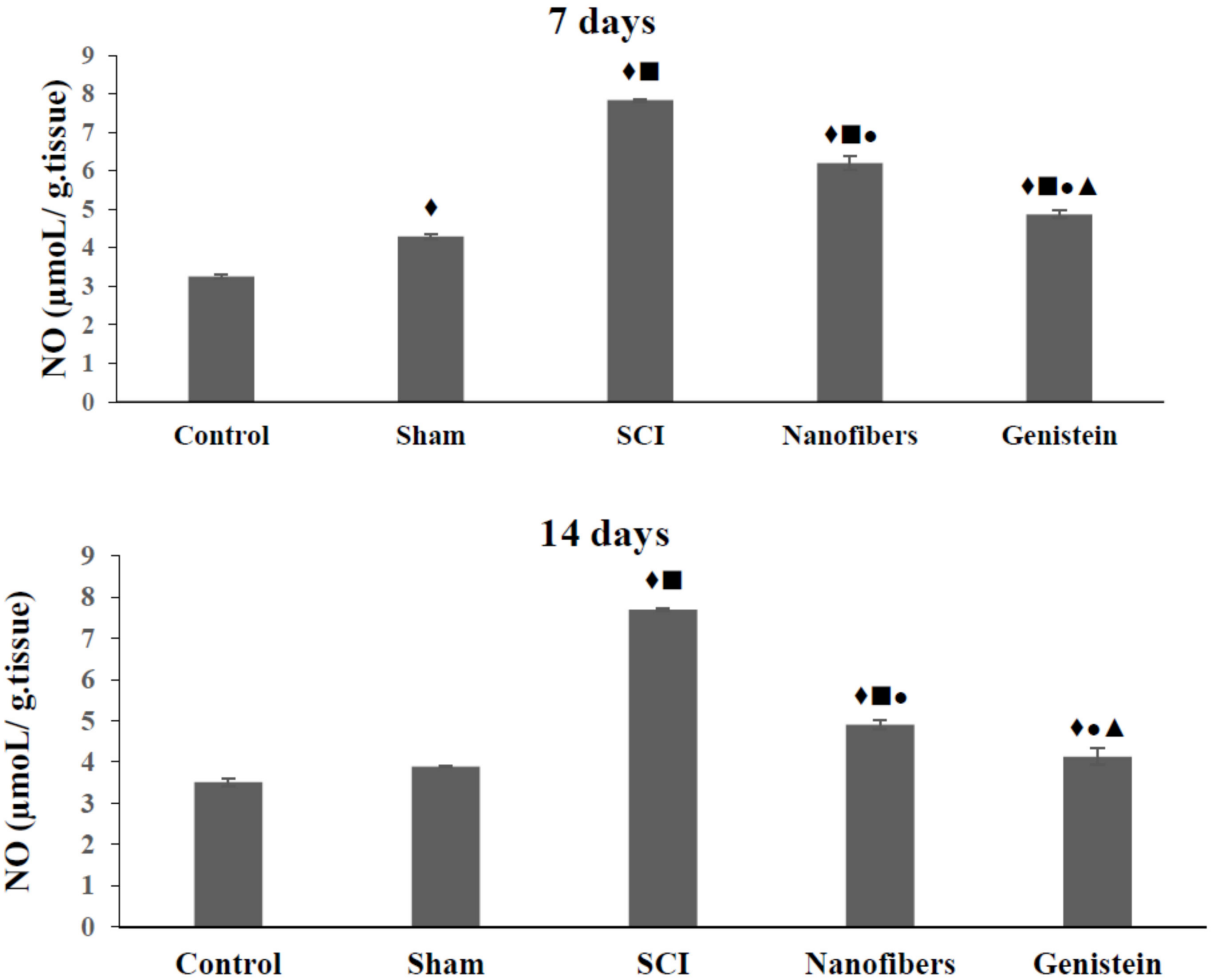
Mean concentration of Nitrous Oxide (NO) (μmol/g tissue) in spinal cord tissue. Nitrous oxide levels were significantly elevated in the injury group when compared with control (♦) and sham (■) groups. Treatment with nanofibers and genistein nanofibers led to a significant (●) decrease of NO levels when compared with spinal cord injury (SCI) group. Genistein nanofibers showed significant reduction in NO as compared to nanofibers (▲) group. Number of animals (*n* = 5). (Mean ± S.E.M.).

Nitrous Oxide (NO) levels (Figure 2) increased significantly (*p* < 0.05) in the SCI group as compared with the control and sham groups. A significant (*p* < 0.05) decrease was recorded in the level of NO in nanofibers and genistein groups as compared to SCI group at all of the time intervals of the experiment. Moreover, the treatment of animals in the SCI group with genistein nanofibers ameliorated the NO level in injured spinal tissue and this amelioration was more pronounced after 14 days of injury.

### 2.3. Genistein Decreased Lipid Peroxidation and Tissue Damage in Spinal Cord Tissue

**Figure 3:**
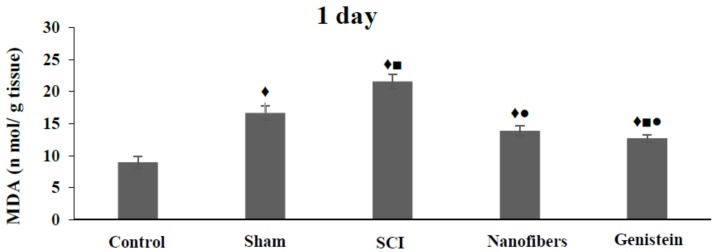

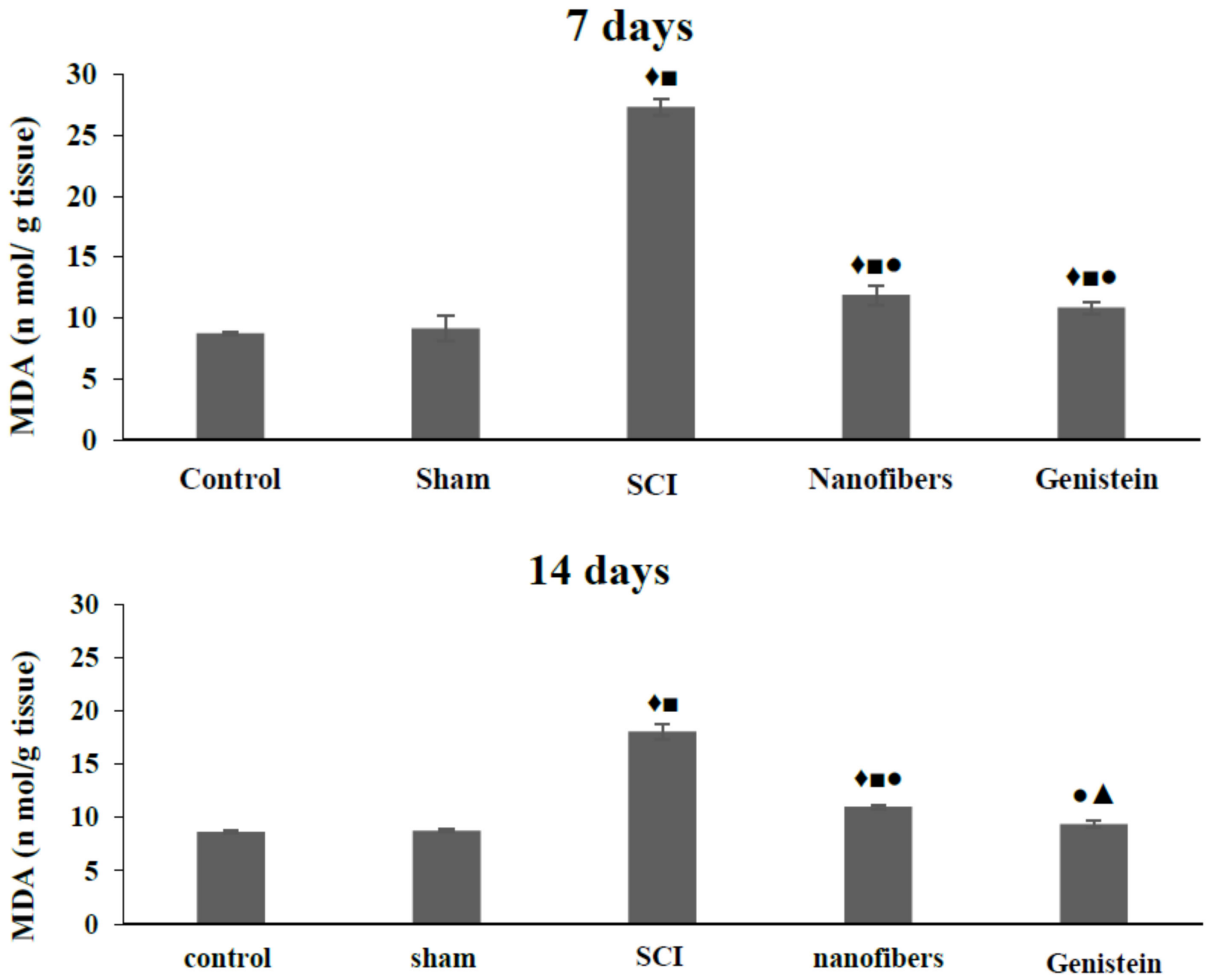
Mean concentration of Malondialdehyde (MDA) (n mol/g tissue) in spinal cord tissue. Spinal cord injury leads to significant (♦, ■, *p* < 0.05) elevation of MDA levels at all time points. Implantation of nanofibers resulted in a significant (●, *p* < 0.05) drop in the level of MDA when compared to SCI group during the whole experimental time. Treatment with genistein nanofibers caused a significant (▲, *p* < 0.05) decrease in MDA after 14 days of injury, which was not significantly different when compared with either control or sham groups. Number of animals (*n* = 5). (Mean ± S.E.M.).

SCI leads to a significant (*p* < 0.05) increase in the Malondialdehyde (MDA) levels (Figure 3), when compared with the control and sham groups at all of the time intervals studied. Implantation of nanofibers only resulted in a significant (*p* < 0.05) drop in the level of MDA when compared to SCI group during the whole experimental time. However, the MDA level remained significantly (*p* < 0.05) higher than the control values throughout the experimental period. Moreover, treatment with nanofibers that were loaded with genistein caused a significant (*p* < 0.05) decrease in MDA levels when compared to the SCI group at the same time points. The level of MDA in spinal cord tissue in genistein group returned to near control level after 14 days of injury, where there was no significant change in its level as compared to either control or sham group.

### 2.4. Interleukin 10 (IL-10) Level in Spinal Cord Tissue

**Figure 4:**
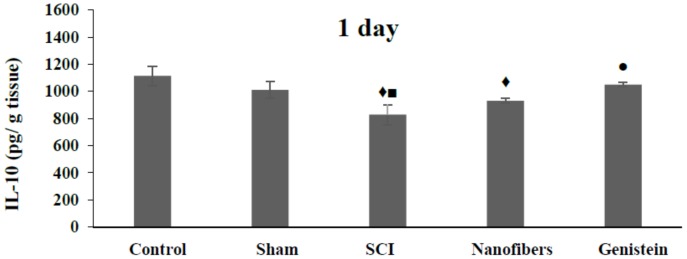

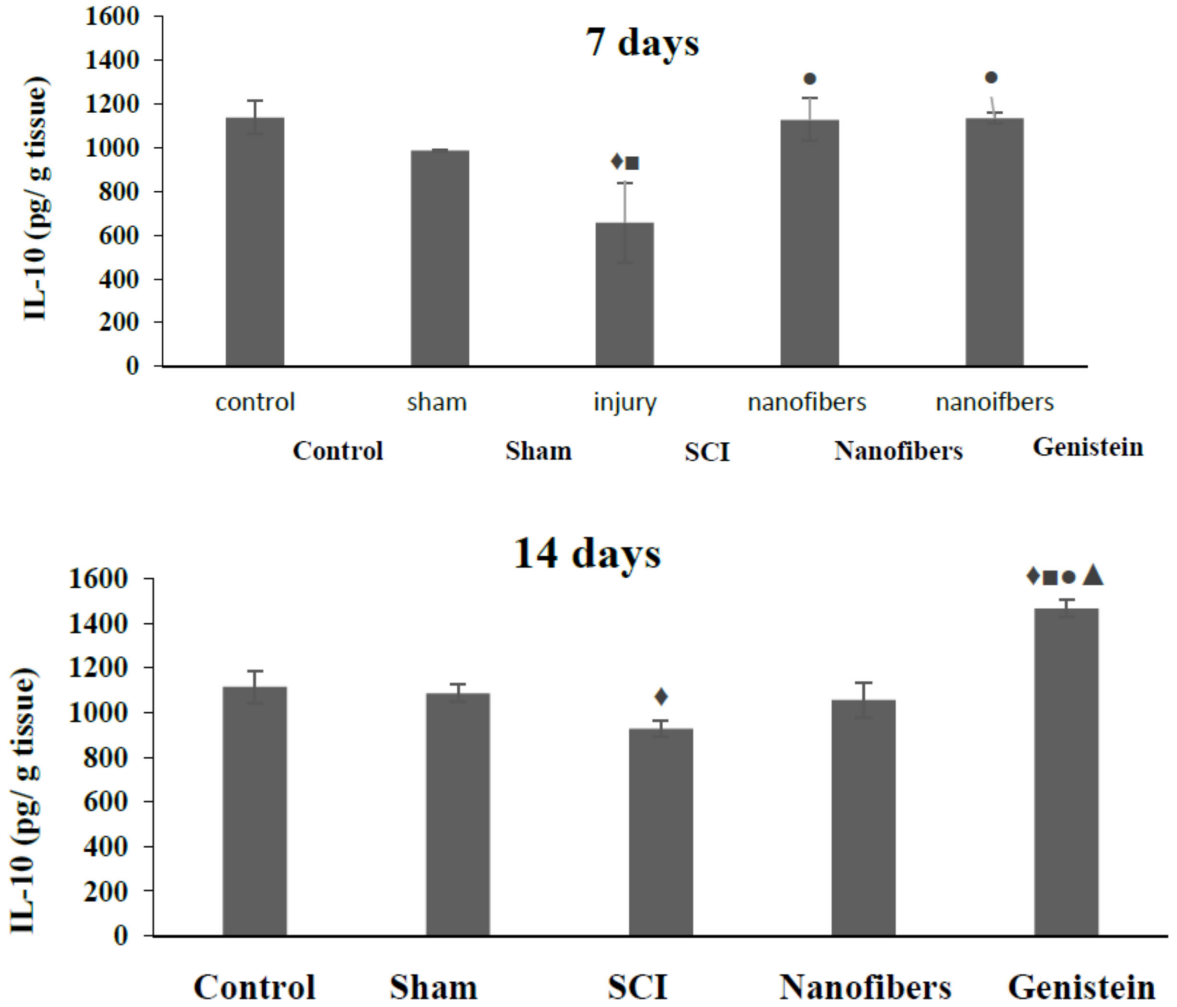
Level of interleukin-10 (IL-10) (pg/g tissue) in spinal cord tissue. Spinal cord injury (SCI) induced a significant (*p* < 0.05) decrease in the IL-10 levels in spinal cord tissue at all the time intervals examined when compared with control group (♦). IL-10 levels were also reduced in the injury group at one and seven days compared to the sham group (■). Nanofibers caused an increase in IL-10 in spinal cord tissue, which was only significant (*p* < 0.05) after seven days with respect to SCI group. On the other hand, treatment with genistein nanofibers induced a significant (*p* < 0.05) elevation in IL-10 level in spinal cord tissue when compared with SCI group (●). Moreover, the increase in IL-10 level was more pronounced at 14 days post injury where it was significant (▲, *p* < 0.05) in comparison to the other four groups. Number of animals (*n* = 5). (Mean ± S.E.M.).

SCI induced a significant (*p* < 0.05) decrease in the IL-10 levels (Figure 4) in spinal cord tissue at all of the time intervals examined when compared with control group. IL-10 levels were also reduced in the injury group at one and seven days compared to the sham group. Nanofibers caused an increase in IL-10 in spinal cord tissue, which was only significant (*p* < 0.05) after seven days with respect to SCI group. On the other hand, treatment with genistein nanofibers induced a significant (*p* < 0.05) elevation in IL-10 level in spinal cord tissue when compared with SCI group. Moreover, the increase in IL-10 level was more pronounced at 14 days post injury where it was significant (*p* < 0.05) in comparison to the other four groups.

### 2.5. Tumor Necrosis Factor—α (TNF-α) Levels in Spinal Cord Tissue

**Figure 5:**
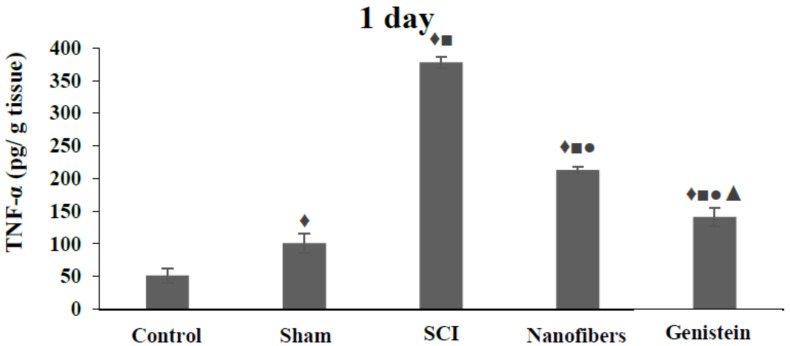

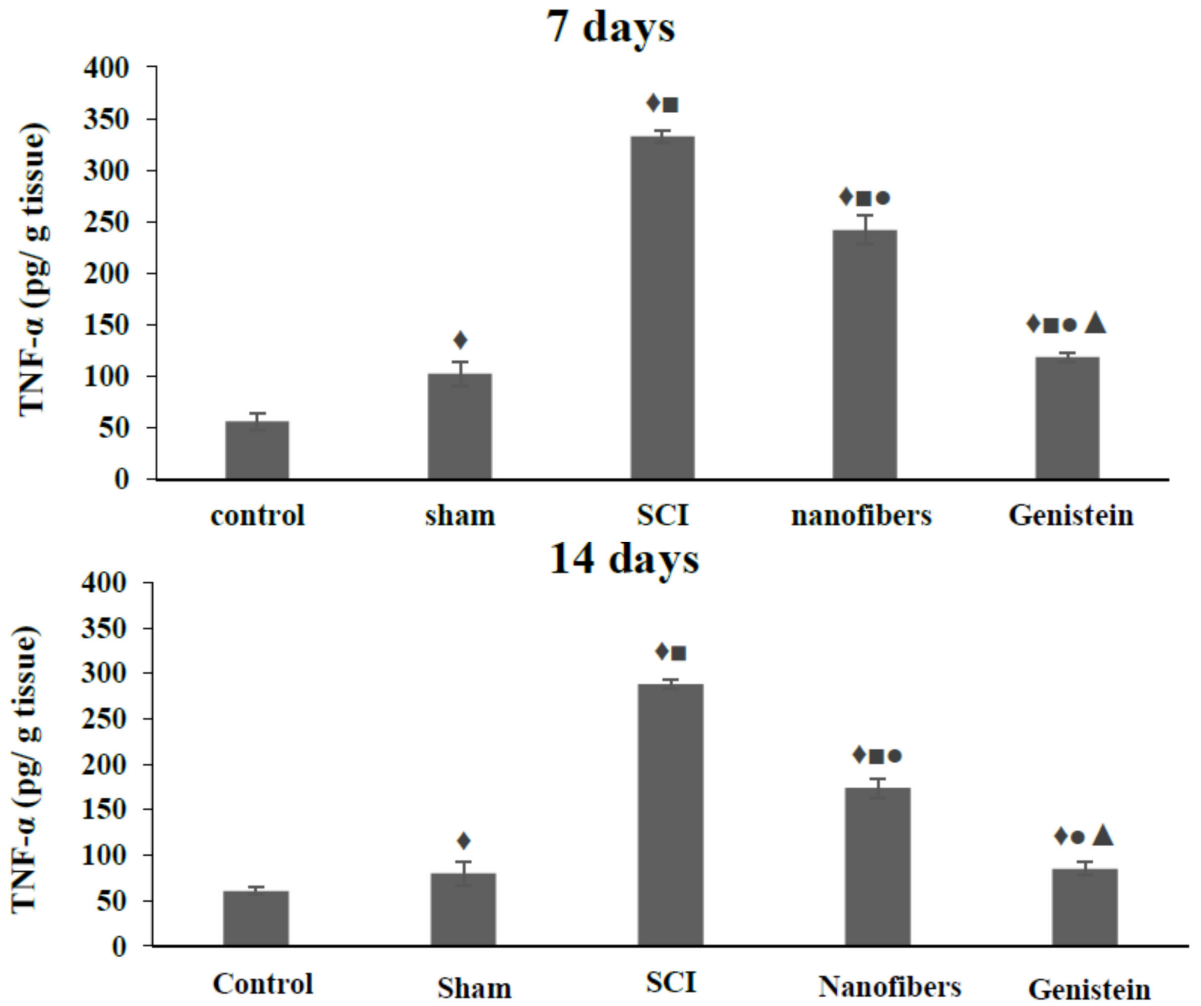
Levels of tumor necrosis factor-alpha (TNF-α) (pg/g tissue) in spinal cord tissue. The TNF-α levels in spinal cord tissue of SCI group exhibited a significant (♦, ■ *p* < 0.05) increase with respect to control and sham groups at all the time intervals. Treatment with nanofibers only or genistein nanofibers caused a significant (●, *p* < 0.05) decrease in the levels of TNF-α in spinal cord tissue at all time points compared to SCI group. Levels of TNF-α in spinal cord tissue decreased significantly (▲, *p* < 0.05) in genistein group when compared with nanofibers group at the same time points. Number of animals (*n* = 5). (Mean ± S.E.M.).

The TNF-α level in spinal cord tissue of the SCI group exhibited a significant (*p* < 0.05) increase with respect to control and sham groups at all of the time intervals investigated (Figure 5). However, treatment of animals with either nanofibers only or nanofibers that were loaded with genistein caused a significant (*p* < 0.05) decrease in the levels of TNF-α in spinal cord tissue at the three time intervals studied when compared with the SCI group. Moreover TNF-α levels in spinal cord tissue decreased significantly (*p* < 0.05) in the genistein treated group as compared to the nanofiber group at the same time points.

## 3. Discussion

Secondary SCI is the result of a group of internal cascade of self-destructive phenomena within the nervous tissue, including lipid hydrolysis, lipid peroxidation, and damage that is caused by hydroxyl radicals [31,32]. In addition, it is recognized that after SCI, the disruption of blood-spinal cord barrier is a key event that leads to inflammation and oxidative stress, causing tissue damage and neurological deficit [33].

The present study provides a novel approach to controlling the secondary damage and reducing tissue damage following experimental SCI. Our results show the beneficial effects of genistein and CS/PVA nanofibers in reducing lipid peroxidation, oxidative damage, and inflammatory response when applied locally to the spinal cord following injury.

SOD has been reported [33,34] to neutralize oxygen-free radicals and protect cells from oxidation by superoxide toxicity. Our current results indicate a significant decrease in the antioxidant enzyme SOD levels following SCI in rats. Previous studies have been also recorded a decrease in the activity of SOD post SCI and attributed this decrease to the extensive presence of free radicals in damaged spinal cord [35,36].

Nitrous oxide (NO) is an endothelium-derived factor that is involved in secondary damage, the increased production of NO causes further neuronal damage and it is considered to be a major regulator of CNS damage. The present result indicating an increase of NO level in SCI group agrees with other researchers [35].

Following CNS trauma, pro-inflammatory cytokines lead to activation of the inducible nitric oxide synthase (iNOS). Production of NO is then increased in injured neuronal tissue. The study of Jiang et al. [35] showed that protein levels and endothelial nitric oxide synthase (eNOS) activity, together with NO concentration were all increased in SCI, subsequently aggravating the damage following SCI. Treatment with genistein nanofibers proved effective in reducing NO levels to almost pre-injury levels, which may contribute to a protective effect and the reduction of neuronal loss in injured spinal cord tissue.

Lipid peroxidation and oxygen free radicals induce oxidative stress, contributing to the pathogenesis of secondary SCI [37,38]. MDA is an end product of the metabolism of unsaturated fatty acid peroxidation [38]. MDA levels reflect the degree of lipid peroxidation and the level of tissue damage after free radical exposure. The present study showed a significant increase in MDA levels in the spinal tissue post SCI, when compared to control and sham groups, indicating that the tissue damage in SCI is partially due to the disruption of oxidant-antioxidant balance. Genistein and nanofiber treatment significantly reduced MDA levels, indicating a possible protective role for both in CNS injuries.

Chronic inflammation is a known event in the secondary damage sequence that follows SCI. In the present study we show a decrease in the level of the anti-inflammatory cytokine IL-10 and an increase of the pro-inflammatory cytokine TNF-α levels in spinal cord tissue following SCI. Both cytokines were significantly changed, indicating a strong anti-inflammatory role for both genistein and CS/PVA nanofibers. This is in agreement with the literature [4,39].

A novel aspect of the present study was to examine the effect of an implantable drug-delivery system in rat post SCI. Nanofibers that are based on the electrospun CS/PVA blends with or without genistein, were shown to improve most of the above mentioned injury-induced changes.

Bio-scaffolds are a promising drug delivery method as they could provide supporting scaffolds for growing cells and tissues [40,41,42,43,44]. They also represent a three-dimensional (3D) environment for axonal growth and migration, which could be modified to simulate the native extracellular matrix [45,46].

Therefore, the scaffold that was used in the present study is not only a space filling agent, but it can also serve a protective role as bioactive molecule delivery systems [39]. CS/PVA nanofibers might have also provided a sustained release of genistein at the injury site for the study period, therefore preventing the need for repeated drug administration.

One of the main objectives of the present work was to evaluate the effects of genistein loaded on nanofibers as implantable drug-delivery system and scaffold. In light of the current data, the treatment of SCI rats with genistein ameliorated all of the investigated parameters. Increase in the activity of the anti-oxidant enzyme SOD and the level of the anti-inflammatory cytokine IL-10, while it caused a decrease in the levels of the neurotransmitter NO, the oxidative stress marker MDA, and the pro-inflammatory cytokine TNF-α in injured spinal cord tissue.

These changes in the studied parameters shifted them toward control values, thereby restoring the balance in the spinal cord. Even though, the levels were still lower or higher than the pre-injury values, the present data confirms previous reports that genistein possibly has a suppressive role in the oxidative stress and inflammatory response. Genistein has been shown to be a strong antioxidant that removes toxic hydroxyl radicals and other ROS that cause lipid peroxidation and DNA and protein damage [10,47].

McClain et al. [48] reported that “genistein, a major natural phytoestrogen in soybean, has a weak estrogenic effect. It has lower binding affinity for estrogen receptor alpha (ERα) than ERβ”, and it therefore lacks unwanted ERα agonist side effects, such as cancer promotion [49]. Others [50], found that genistein also has effects that are non-dependent on its estrogen-like activity, including protein tyrosine kinase inhibition or down-regulation, immune system modulation, and anti-oxidant activity. Liu et al., [51] showed that genistein can cross the Blood Brain Barrier (BBB) reaching the CNS. It is worth noting that almost all the nervous system cells and immune cells all have ERβ receptors [49,52,53,54].

In conclusion, the treatment of rats post SCI with CS/PVA nanofibers (with or without genistein) improved most of the injury-induced changes in the investigated biochemical parameters and shifted them toward the control group values. The combination of bio-scaffolds and genistein is a promising therapeutic combination for treating inflammatory conditions that follow CNS trauma.

## 4. Materials and Methods

### 4.1. Preparation of CS/PVA Nanofibers

**Figure 6:**
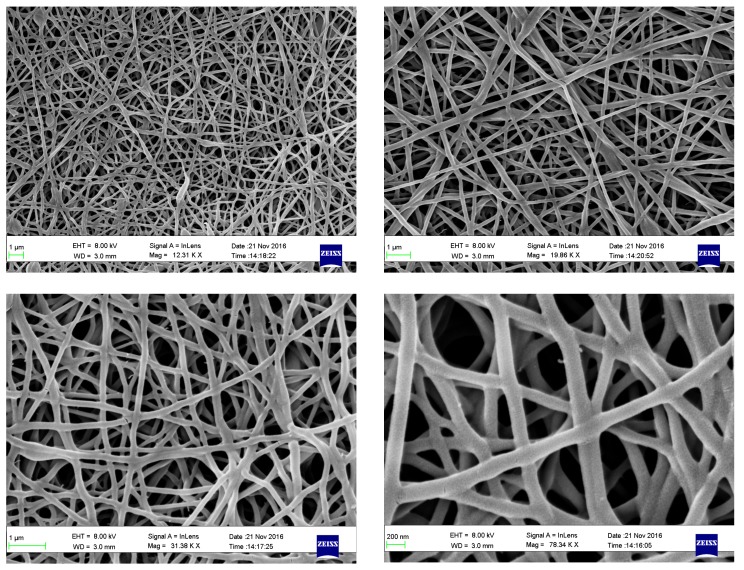
Scanning electron micrograph of nanofibers, showing uniform structure and diameter of nanofibers. We previously [54] reported the optimum conditions for preparation of chitosan/polyvinyl alcohol (CS/PVA) nanofibers (volume ratio 30/70 CS/PVA, concentration of mixture 50%, voltage 25 KV, flow rate 0.7 mL/h., and tip-to-collector distance (TCD) 10 cm.

Electrospinning was used to fabricate CS/PVA nanofibers using acetic acid/distilled water solution mixture as a solvent [40]. We have previously described the fabrication and characterization of nanofibers that are loaded with genistein [55]. Briefly, optimum preparation conditions for CS/PVA nanofibers (Figure 6), were established, as follows; polymers volume ratio 30/70 CS/PVA, concentration of mixture 50%, applied voltage 25 KV, flow rate 0.7 mL/h, and tip to collector distance (TCD) 10 cm, followed by physical crosslinking [55].

We previously reported [55] the drug release from nanofibers by immersing a (1 × 1 cm^2^) drug loaded nanofibers into 100 mL phosphate buffer solution (PBS), at 37 °C. Samples were removed from the medium at 0.5, 1, 4, 8 and 24 h, and the concentration of the drug was determined by spectrophotometry at a λ max of 280 nm.

Cytotoxicity of crosslinked CS/PVA nanofibers was previously described while using MTT assay (ATCC^®^ 30-1010K) on human fibroblast cells (ATCC CCL-75 W1 38) [55].

### 4.2. Experimental Animals

#### 4.2.1. Handling of Animals

A total of 75 adult female Sprague-Dawley rats (RRID: MGI:5651135) (weighing ≈ 200–250 g) were housed in polypropylene cages in climate controlled rooms, with standard food pellets and drinking water ad libitum. All of the surgical procedures and post-surgical care were performed in compliance with the national institute of health (NIH) guidelines for the Care and Use of Laboratory Animals.

All experiments in the present study were conducted in compliance with the guidelines established by the Institutional Animal Care and Use Committee (IACUC) of Cairo University (CU-IF-90-17, 1 November 2017).

#### 4.2.2. Surgical Procedure of Spinal Cord Injury

Female Sprague-Dawley rats were randomized using block randomization method, into five groups. Group (1) Control. Group (2) Sham control group: the skin was prepared before incision, laminectomy (excision of a vertebral lamina) only with no injury of the spinal cord. Group (3) SCI group: (laminectomy + SCI) Laminectomy with right lateral hemi-section SCI at the T 9–10. Group (4) Nanofibers group: (laminectomy + SCI + nanofibers) laminectomy with right lateral hemi-section SCI at the T 9–10, followed by the immediate application of nanofibers without genistein. Group (5) Genistein group: (laminectomy+ SCI + nanofibers + genistein) laminectomy with right lateral hemi-section cord injury at the T 9–10, followed by the immediate application of nanofibers that were loaded with genistein. Ketamine anesthesia (75 mg/kg intraperitoneal), was used according to IACUC guidelines. Skin was prepped and incised at the back of rats in treated groups, muscles were split, and laminectomy was performed under dissecting microscope at the T 9–10 vertebral level, the cord was exposed, and the dura was incised and pulled laterally. Spinal cord hemisection using micro iris scissors was made at T 9–10, followed by placement of nanofibers in group 4 and application of nanofibers loaded with genistein in group 5, then the dura was sutured, and muscles and skin were closed in layers. All animals received post-operative analgesia (ketoprofen 5 mg/kg SC/24 h) for three days. Animals were monitored daily for the duration of the experiment for signs of pain and distress e.g., back arching, vocalization, and analgesia was administered accordingly. For SCI animals, manual bladder expression was performed 2–4 times/day and sutures were removed seven days postoperatively.

**Figure 7:**
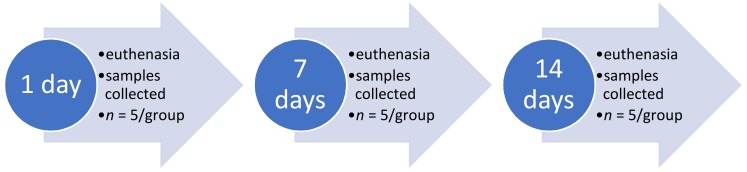
Flow chart showing the experimental timeline.

Animals were randomly assigned to either control or experimental groups (sham, injury, injury and genistein nanofibers, injury, and nanofibers). Control group was euthanized immediately and the cord samples collected while other groups were euthanized at days 1, 7, or 14, where five animals from each group were euthanized and samples collected, the flow chart (Figure 7) shows the experimental timeline.

Researchers were blinded to the experimental groups. There were no sample size differences between the beginning and end of the experiments, animals were added to replace animals lost due to morbidity or mortality. Animals showing signs of morbidity, such as infection at the injury site or other diseases were excluded from the study and replaced. Animals that were healthy and showing no signs of infection were included in the study. At 1, 7, and 14 days post-surgery, rats were sacrificed with an overdose of pentobarbital (Thiopental sodium) 75 mg/kg, IP. All the specimens of spinal cord were plotted dry and weighed, and 0.025 g of each specimen homogenized in 1 mL of 0.1 M phosphate buffer solution (PBS) (pH 7.4). The homogenate was centrifuged at 3000 rpm for 20 min at 4 °C and the supernatant was aliquoted and stored at −80 °C until use.

### 4.3. Biochemical Analyses

#### 4.3.1. Determination of Super Oxide Dismutase (SOD) Activity

The activity of anti-oxidative enzyme super oxide dismutase (SOD) was determined using calorimetric assay (Biodiagnostic, Giza, Egypt, CAT. No. SD 25 21), according to the method that was originally described by Nishikimi, et al. [56]. The assay is based on the ability of SOD in the tested sample to inhibit phenazine methosulphate-mediated reduction of nitroblue tetrazolium dye [56].

#### 4.3.2. Determination of Nitrous Oxide (NO) Concentration

The level of the NO was estimated in spinal cord tissue by using calorimetric assay (CAT. No. NO 25 33 Biodiagnostic, Giza, Egypt), according to the method that was originally described by Montgomery et al. [57]. In acid medium and in the presence of nitrite, the formed nitrous acid diazotise sulphanilamide and the product is coupled with *N*-(1–naphthyl) ethylenediamine. The color intensity of the resulting azo dye was measured by spectrophotometry at 540 nm [57].

#### 4.3.3. Determination of MDA Concentration

The oxidative stress marker malondialdehyde (MDA) was determined in the spinal cord tissue homogenate by using Thiobarbituric acid (TBA) calorimetric assay method, according to Ohkawa et al. [58] (Biodiagnostic, Giza, Egypt, CAT. No. MD 25 29).

#### 4.3.4. Quantitative Determination of IL-10 and TNF-α by Enzyme-Linked Immunosorbent Assay (ELISA)

The anti-inflammatory cytokine IL-10 (CAT. No. K0331123HS, KOMA BIOTECH, Seoul, Korea), and the pro-inflammatory cytokine TNF-α were quantitatively determined (CAT. No. K0331196, KOMA BIOTECH, Seoul, Korea) in the spinal cord tissue of rats by the ELISA technique, according to the manufacturer’s instructions.

#### 4.3.5. Statistical Analyses

Data points at 14 days were considered end points for the current experiment. Data sets were assessed for normality using SPSS^®^ and data points outside 95% confidence intervals were considered to be outliers and excluded from analysis.

The statistical analyses were carried out using SPSS^®^ version 15 software. All data were expressed as mean ± standard error of mean (S.E.M.). The independent variables of individual comparisons were illustrated by using Least Significant Difference (LSD) post-hoc test of one-way ANOVA to compare the differences of mean values between different groups. *P* values that were less than 0.05 are considered to be statistically significant.

## Abbreviations

ANOVA	Analysis of variance
BBB	Blood brain barrier
CS	Chitosan
CNS	Central nervous system
eNOS	endothelial nitric oxide synthase
iNOS	inducible nitric oxide synthase
IACUC	Institutional Animal Care and Use Committee
IL-10	interleukin 10
IP	intraperitoneal
KV	Kilo volt
LSD	Least Significant Difference
MDA	Malondialdehyde
NIH	National institute of health
NO	Nitrous oxide
PBS	phosphate buffer solution
PVA	Polyvinyl alcohol
ROS	reactive oxygen species
SCI	Spinal cord injury
S.E.M	standard error of mean
SOD	superoxide dismutase
TCD	tip-to-collector distance
TNF-α	tumor necrosis factor-α
